# Prognostic impact of lymph node metastasis along the left gastric artery in esophageal squamous cell carcinoma

**DOI:** 10.1186/s13019-021-01466-2

**Published:** 2021-05-03

**Authors:** Xuan Liu, Leilei Wu, Dongkun Zhang, Peng Lin, Hao Long, Lanjun Zhang, Guowei Ma

**Affiliations:** 1 Department of thoracic surgery, State Key Laboratory of Oncology in South China, Collaborative Innovation Center for Cancer Medicine, Sun Yat-sen University Cancer Center, Guangdong Esophageal Cancer Institute, 651 Dongfengdong Road, Guangzhou, 510060 China; 2 Department of Thoracic Surgery, Guangdong Provincial People’s Hospital/Guangdong Academy of Medical Sciences, Guangzhou, 510080 China

**Keywords:** Esophageal squamous cell carcinoma, Left gastric artery, Lymph node metastasis, Survival

## Abstract

**Background:**

Although the incidence of lymph node (LN) metastasis (LNM) along the left gastric artery is high, its relationship with the prognosis in postoperative patients with esophageal squamous cell carcinoma (ESCC) is rarely reported. This study clarified the prognostic impact of LNM along the left gastric artery in postoperative patients with ESCC.

**Methods:**

This study assessed data of 1521 patients with ESCC who underwent esophagectomy at the Sun Yat-sen University Cancer Center between March 1992 and March 2012. A chi-squared test and Mann-Whitney *U* test were used to explore the preliminary correlation between clinical factors and LNM along the left gastric artery. Univariate and multivariate Cox regression analyses were used to assess whether LNM along the left gastric artery was an independent predictor of overall survival. Kaplan–Meier analysis and the log-rank test were used to present a classifying effect based on LN status.

**Results:**

LNM was observed in 598 patients (39.3%) and was found along the branches of the left gastric artery in 256 patients (16.8%). The patients were classified into two groups based on the presence of LNM along the left gastric artery. Patients without LNM along the left gastric artery had better cancer-specific survival than those with positive LNs (*P* <  0.001).

**Conclusions:**

This study indicated that LNM along the left gastric artery was an important independent prognostic factor for long-term survival among ESCC patients (*P* = 0.011).

## Introduction

Esophageal cancer (EC) is one of the most common cancers worldwide, with an estimated 604,100 new cases occurring globally in 2020. It is the sixth most common cause of death from cancer [1]. In China, EC is the fourth most common cause of mortality and is often located in the thorax, while 95% of EC cases are pathologically diagnosed as squamous cell carcinoma [2]. The treatment of esophageal cancer is still surgical resection combined with multimodality therapy, but the overall survival remains unsatisfactory.

Lymph node (LN) metastasis (LNM) is one of the single most important prognostic factors in EC [3]. LN status plays an important role in assessing the conditions of esophageal squamous cell carcinoma (ESCC) patients, including helping determine the dose of chemoradiotherapy or surgical approach, and aids in predicting prognosis [4–7]. Several studies have reported that the number of positive LNs was an independent prognostic indicator of survival in patients with EC [8–13]. The 8th edition of the American Joint Committee on Cancer (AJCC) Staging Manual for EC categorizes the N stage according to the number of metastatic LNs, irrespective of the site [14]. However, the 11th edition of the Japanese Classification of EC categorizes N stage according to both the site and the number of metastatic LNs [15].

Some previous studies indicated that LNM in specific areas, such as subcarinal, thoracic, and recurrent laryngeal nerve LNs, correlated with poor prognosis in ESCC patients [16, 17]. LN dissection around the gastric artery is commonly performed for the surgical management of EC. We found that the incidence of LNM along the left gastric artery is high; however, its relationship with prognosis is rarely reported. This study aimed to perform a retrospective analysis to determine whether LNM presence along the left gastric artery was associated with decreased survival after esophagectomy in ESCC patients.

## Methods

### Patients

The study protocol was approved by the Ethics Committee of Sun Yat-sen University Cancer Center (approval no. YB2016–070). Due to the retrospective nature of this study, all data were de-identified and the need for written informed consent was waived. We assessed the data of 1521 patients with ESCC who underwent surgical treatment at the Department of Thoracic Surgery at Sun Yat-sen University Cancer Center between March 1992 and March 2012. Our department conducted standard surgical procedures, including Ivor Lewis, McKeown, and Sweet. And the strategy of lymph node dissection is based on both the location of the tumor and the surgeon’s preference, all patients were treated with thoracoabdominal lymph node dissection. Patients who underwent neoadjuvant and adjuvant therapy were also included. Neoadjuvant and adjuvant therapy are recommended according to the guidelines of our center and the guidelines of China. All tumors were located at the thoracic esophagus.

**Fig. 1:**
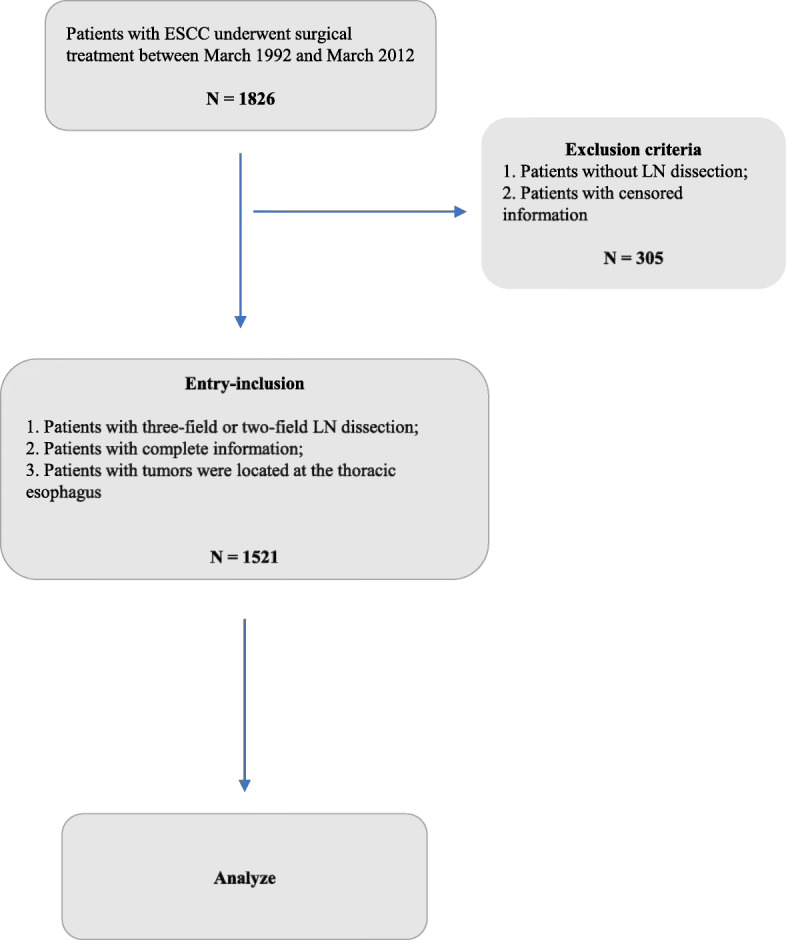
Flow chart of this study

We extracted the following data for each patient from the medical records: age, sex, tumor length, tumor location, differentiation, T stage (depth of invasion), vessel involvement, LNM, number of positive LNs along the left gastric artery, and N stage. All patients were staged according to the 8th edition of the AJCC staging manual for EC. Moreover, we classified patients into two groups based on the presence of LNs metastasis along the left gastric artery. The flow chart of patient selection is shown in Fig. 1.

### Follow-up

The patients were followed up at our outpatient department every 3 months for the first 2 years, every 6 months for the next 3 years, and annually thereafter. The data were updated with the latest findings. During follow-up, clinical histories were obtained, and the patients underwent physical examination, routine blood examination, barium swallow, chest radiography, abdominal ultrasonography, cervical ultrasonography, and/or computed tomography (CT) scans from the neck to the abdomen. Patients experiencing tumor-related pain or other clinical symptoms indicating distant metastasis were recommended to undergo positron emission tomography (PET-CT).

### Statistical analysis

All potential and reported predictive factors associated with LNM, including demographic data and tumor characteristics, were analyzed using the chi-squared test or Mann–Whitney *U* test, as appropriate. Significant factors were extracted for further analysis. Cancer-specific survival (CSS) curves and overall survival (OS) curves were analyzed using the Kaplan–Meier method, and the log-rank test was used to estimate prognostic values. Univariate and multivariate analyses of survival were performed using Cox regression to estimate hazard ratios (HR) with 95% confidence intervals (CIs) and to identify independent prognostic factors. All statistical analyses were performed using the SPSS version 25.0 (IBM Corp., Armonk, NY, USA). Analysis items with *P* <  0.05 were considered statistically significant.

## Results

### Clinicopathological characteristics

The clinicopathological characteristics of the 1521 patients are shown in Table 1. Overall, 1174 men and 347 women with a median age of 58.0 (range, 28–88) years were enrolled in the study. Among these patients, 998 (65.6%) had a history of smoking and 531 (34.9%) had a history of drinking. In addition, 33 (2.2%) patients had vascular tumor thrombi. In most cases (775/1521, 50.9%) tumors were located at the mid-thoracic esophagus. Most patients (1408/1521, 92.6%) underwent two-field LN dissection and others underwent three-field LN dissection. Meanwhile, 112 patients had subcarinal LNM and 20 patients had only subcarinal LNM; 117 patients had recurrent laryngeal nerve LNM and 48 patients had only recurrent laryngeal nerve LNM. Moreover, 73 (4.8%) patients received neoadjuvant therapy and 243 (16.0%) underwent postoperative adjuvant therapy.

**Table 1:** Clinicopathological characteristic of patients

	All cases (1521, %)	LN metastasis along the left gastric artery (256, %)
Age (years)		
≤ 60	**938 (61.7)**	**163 (63.7)**
> 60	**583 (38.3)**	**93 (36.3)**
Gender		
Male	**1174 (77.2)**	**227 (88.7)**
Female	**347 (22.8)**	**29 (11.3)**
Smoking history		
Yes	**998 (65.6)**	**186 (72.7)**
No	**523 (34.4)**	**70 (27.3)**
Drinking history		
Yes	**531 (34.9)**	**113 (44.1)**
No	**990 (65.1)**	**143 (55.9)**
Differentiation		
Well	**388 (25.5)**	**54 (21.1)**
Moderate	**711 (46.7)**	**106 (41.4)**
Poor/un-	**422 (27.7)**	**96 (37.5)**
Vascular invasion		
Yes	**33 (2.2)**	**14 (5.5)**
No	**1488 (97.8)**	**242 (94.5)**
Tumor location		
Upper thoracic esophagus	**255 (16.8)**	**11 (4.3)**
Middle thoracic esophagus	**775 (50.9)**	**117 (45.7)**
Lower thoracic esophagus	**491 (32.3)**	**128 (50.0)**
Tumor length		
< 3	**396 (26.0)**	**44 (17.2)**
3–5	**890 (58.5)**	**161 (62.9)**
> 5	**235 (15.5)**	**51 (19.9)**
T stage		
Tis	**30 (2.0)**	**0**
T1	**147 (9.7)**	**16 (6.3)**
T2	**349 (22.9)**	**36 (14.1)**
T3	**961 (63.2)**	**189 (73.8)**
T4	**34 (2.2)**	**15 (5.9)**
N staging		
N0	**923 (60.7)**	**0**
N1	**340 (22.4)**	**108 (42.2)**
N2	**199 (13.1)**	**106 (41.4)**
N3	**59 (3.9)**	**42 (16.4)**
AJCC 8th stage		
Stage 0	**30 (2.0)**	**0**
Stage I	**193 (12.7)**	**0**
Stage II	**715 (47.0)**	**14 (5.5)**
Stage III	**507 (33.3)**	**192 (75.0)**
Stage IV	**76 (5.0)**	**50 (19.5)**
Neoadjuvant therapy		
Yes	**73 (4.8)**	**12 (4.7)**
No	**1448 (95.2)**	**244 (95.3)**
Postoperative adjuvant therapy		
Yes	**243 (16.0)**	**82 (32.0)**
No	**1278 (84.0)**	**174 (68.0)**
Subcarinal LN metastasis		
Yes	**112 (7.4)**	**49 (19.1)**
No	**1409 (92.6)**	**207 (80.9)**
Recurrent laryngeal nerve LN metastasis		
Yes	**117 (7.7)**	**24 (9.4)**
No	**1404 (92.3)**	**232 (90.6)**
Left gastric artery LN metastasis		
Yes	**256 (16.8)**	
No	**1265 (83.2)**	
Number of resected TLNs		
Mean ± SD	**20.98 ± 13.54**	**22.19 ± 11.62**
Median (minimum, maximum)	**18.00 (1, 109)**	**19.00 (5, 87)**

Displayed the clinicopathological characteristic of patients with Esophageal Squamous Cell Carcinoma (ESCC), and showed the proportions in all the subjects and in left gastric artery lymph node metastasis of these factors.

*LN* Lymph node, *TLNs* Total lymph node.

### Patients with LNM along the left gastric artery

LNM was observed in 598 patients (39.3%) and was found along the left gastric artery in 256 patients (16.8%) (Table 1). There were 23 patients with cervical LNM, 436 patients with chest LNM, and 348 patients with upper abdomen LNM (Table 2). We classified the patients into two groups (0 and 1 groups) according to the presence or absence of LNs metastasis of LNs metastasis along the left gastric artery as 1265 (83.2%) and 256 (16.8%) patients, respectively.

**Table 2:** Details of LNM and TNM stage

	0 (30, %)	I (193, %)	II (715, %)	III (507, %)	IV (76, %)	Total	*P* value
LNM in thorax							**< 0.001**
No	**30 (100.0)**	**193 (100.0)**	**706 (98.7)**	**147 (29.0)**	**9 (11.8)**	**1085**	
Yes	**0 (0.0)**	**0 (0.0)**	**9 (1.3)**	**360 (71.0)**	**67 (88.2)**	**436**	
LNM in upper abdomen							**< 0.001**
No	**30 (100.0)**	**193 (100.0)**	**700 (97.9)**	**234 (46.2)**	**16 (21.1)**	**1173**	
Yes	**0 (0.0)**	**0 (0.0)**	**15 (2.1)**	**273 (53.8)**	**60 (78.9)**	**348**	
LNM cervical							**< 0.001**
No	**30 (100.0)**	**193 (100.0)**	**714 (99.9)**	**488 (96.3)**	**68 (89.5)**	**1456**	
Yes	**0 (0.0)**	**0 (0.0)**	**1 (0.1)**	**19 (3.7)**	**8 (10.5)**	**23**	
Subcarinal LN metastasis							**< 0.001**
No	**30 (100.0)**	**193 (100.0)**	**714 (99.9)**	**429 (84.6)**	**43 (56.6)**	**1409**	
Yes	**0 (0.0)**	**0 (0.0)**	**1 (0.1)**	**78 (15.4)**	**33 (43.4)**	**112**	
Recurrent laryngeal nerve LN metastasis							**< 0.001**
No	**25 (100.0)**	**171 (100.0)**	**708 (99.0)**	**417 (82.2)**	**56 (73.7)**	**1404**	
Yes	**0 (0.0)**	**0 (0.0)**	**7 (1.0)**	**90 (17.8)**	**20 (26.3)**	**117**	
Left gastric artery LN metastasis							**< 0.001**
No	**25 (100.0)**	**171 (100.0)**	**701 (98.0)**	**315 (62.1)**	**26 (34.2)**	**1265**	
Yes	**0 (0.0)**	**0 (0.0)**	**14 (2.0)**	**37.9 (41.1)**	**50 (65.8)**	**256**	

Displayed the different pattern of LNM in different N stage, and the significance was conducted with Fisher’s exact test.

According to the chi-squared test and Mann–Whitney *U* test results shown in Table 3, smoking history, drinking history, differentiation, vascular invasion, tumor location, tumor length, T stage, N stage, number of resected TLNs were associated with LNM along the left gastric artery. However, the age was not related with LNM along the left gastric artery (Table3).

**Table 3:** Factors association with the left gastric artery lymph node

	NO(*n* = 1265)	YES(*n* = 256)	χ2 / M-W U	*P* value
Age (mean ± SD)	**58.05 ± 9.12**	**57.53 ± 9.04**	−0.584*	**0.559**
Gender			**23.060**^#^	**< 0.001**
Male	**947 (74.86)**	**227 (88.67)**		
Female	**318 (25.14)**	**29 (11.33)**		
Smoking history			**6.765**^#^	**0.009**
Yes	**812 (64.19)**	**186 (72.66)**		
No	**453 (35.81)**	**70 (27.34)**		
Drinking history			**11.539**^#^	**0.001**
Yes	**418 (33.04)**	**113 (44.14)**		
No	**847 (66.86)**	**143 (55.86)**		
Differentiation			**14.787**^#^	**0.001**
Well	**334 (26.40)**	**54 (25.51)**		
Moderate	**605 (47.83)**	**106 (46.75)**		
Poor/un-	**326 (25.77)**	**96 (27.74)**		
Vascular invasion			**15.784**^#^	**< 0.001**
Yes	**19 (1.50)**	**14 (5.47)**		
No	**1246 (98.50)**	**242 (94.53)**		
Tumor location			**60.143**^#^	**< 0.001**
Upper thoracic esophagus	**244 (19.29)**	**11 (4.30)**		
Middle thoracic esophagus	**658 (52.01)**	**117 (45.70)**		
Lower thoracic esophagus	**363 (28.70)**	**128 (50.00)**		
Tumor length (mean ± SD)	**3.70 ± 1.68**	**4.14 ± 1.71**	−3.883*	**< 0.001**
T stage			**43.976**^#^	**< 0.001**
Tis	**30 (2.37)**	**0**		
T1	**131 (10.36)**	**16 (6.25)**		
T2	**313 (24.74)**	**36 (14.06)**		
T3	**772 (61.03)**	**189 (73.83)**		
T4	**19 (1.50)**	**15 (5.86)**		
N stage			**554.208**^#^	**< 0.001**
0	**923 (72.96)**	**0**		
1	**232 (18.34)**	**108 (42.19)**		
2	**93 (7.35)**	**106 (41.41)**		
3	**17 (1.34)**	**42 (16.41)**		
Number of resected TLNs (mean ± SD)	**20.74** ± **13.89**	**22.19** ± **11.62**	−3.155*	**0.002**

Chi-Square and Mann-Whitney U test were used to explore the factors association with the left gastric artery lymph node (LN).

^#^Chi-Square test for ESCC patients grouped with categorical variables, when the theoretical frequency is less than 1, the probability can only be calculated using the Fisher’s exact test

*Mann-Whitney U test for ESCC patients grouped with continuous variables

### Cox proportional hazards regression analysis

Prognostic factors affecting long-term survival are shown in Table 4. Variables with *P* <  0.05 in the univariate analysis were included in the multivariate Cox regression analysis. Multivariate Cox proportional hazards regression analysis revealed that age, differentiation, drinking history, vascular tumor thrombus, T stage, N stage, treatment and left gastric artery metastasis were independent prognostic predictors of survival in ESCC patients.

**Table 4:** Univariate and multivariate analyses with cox regression in patients with ESCC

	Univariate Analyses			Multivariate Analyses		
	***P*** value	HR	95%CI	***P*** value	HR	95%CI
**Age**	**< 0.001**	**1.019**	**(1.011–1.027)**	**< 0.001**	**1.022**	**(1.014–1.030)**
**Gender**	**0.001**	**0.749**	**(0.631–0.888)**	**0.990**	**0.998**	**(0.780–1.278)**
**Differentiation**	**< 0.001**	**1.250**	**(1.138–1.373)**	**0.010**	**1.137**	**(1.030–1.253)**
**Smoking history**	**< 0.001**	**1.327**	**(1.145–1.538)**	**0.425**	**1.092**	**(0.880–1.354)**
**Drinking history**	**< 0.001**	**1.356**	**(1.179–1.558)**	**0.040**	**1.178**	**(1.007–1.377)**
**Vascular tumor thrombus**	**< 0.001**	**2.413**	**(1.632–3.569)**	**0.038**	**1.535**	**(1.023–2.303)**
**Tumor location**	**0.802**	**1.013**	**(0.915–1.122)**			
**T stage**	**< 0.001**	**1.637**	**(1.471–1.821)**	**< 0.001**	**1.417**	**(1.262–1.590)**
**N stage**	**< 0.001**	**1.675**	**(1.559–1.801)**	**< 0.001**	**1.497**	**(1.335–1.679)**
**Tumor length**	**< 0.001**	**1.115**	**(1.073–1.158)**	**0.075**	**1.041**	**(0.996–1.087)**
**Treatment**	**0.024**	**1.111**	**(1.014–1.217)**	**0.040**	**0.901**	**(0.816–0.995)**
**Left gastric artery LN metastasis**	**< 0.001**	**2.177**	**(1.847–2.566)**	**0.011**	**1.092**	**(1.020–1.169)**
**Subcarinal LN metastasis**	**< 0.001**	**2.263**	**(1.814–2.822)**	**0.652**	**1.061**	**(0.819–1.375)**
**Recurrent laryngeal nerve LN metastasis**	**0.009**	**1.364**	**(1.081–1.720)**	**0.233**	**0.850**	**(0.650–1.110)**

Using the Cox regression performed the Univariate and Multivariate Analyses, shown the relation with the prognosis.

The factors in the univariate analyses with *P* value less than 0.05 would be took in account into multivariate analyses.

*ESCC* Esophageal Squamous Cell Carcinoma, *LN* Lymph node, *HR* Hazard ratio, *CI* Confident interval.

### Prognostic analysis

**Fig. 2:**
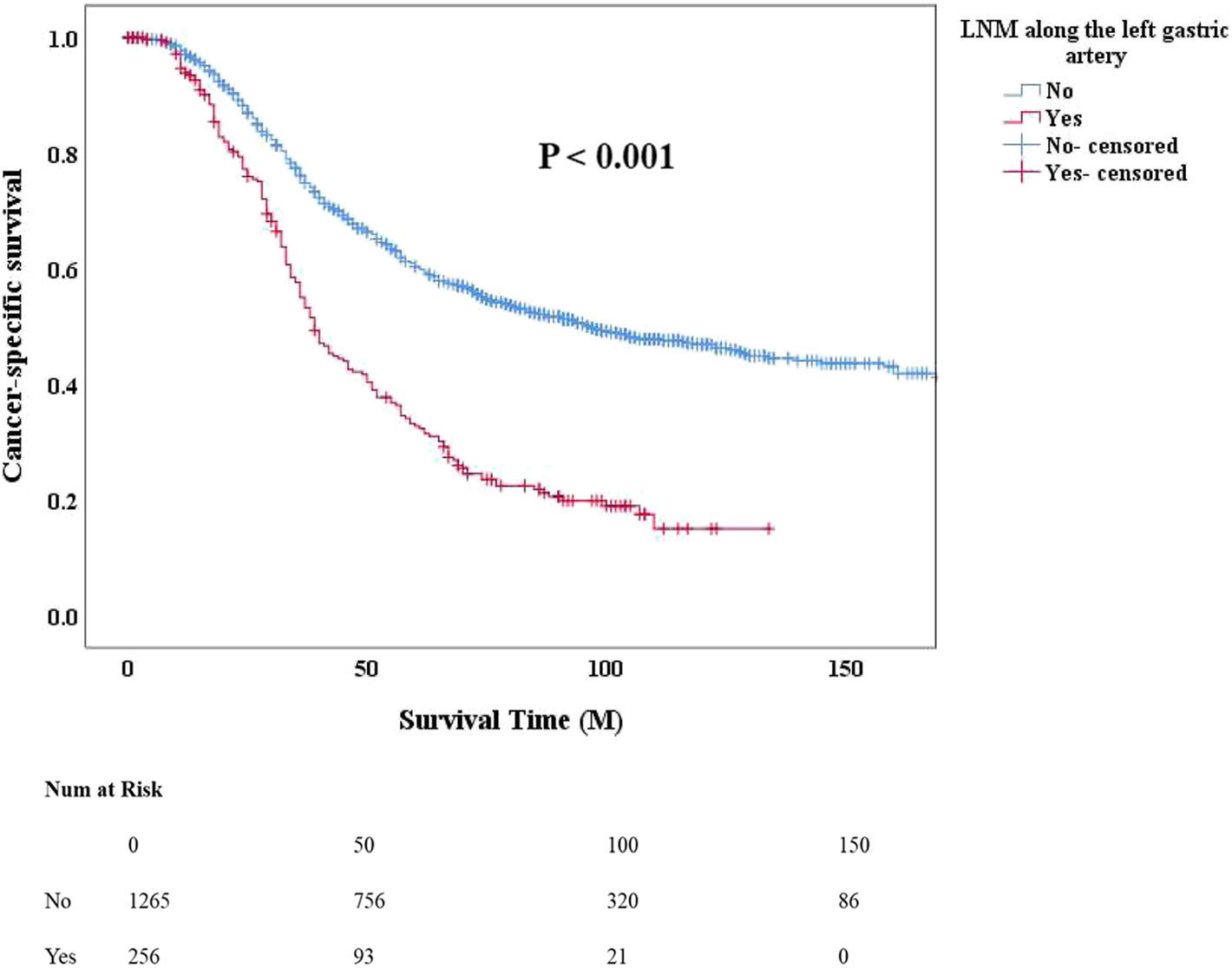
Cancer-specific survival of patients based on whether with left gastric artery LN metastasis. Cancer-specific survival curves were used to compare the prognostic significance between the groups with and without left gastric artery metastasis. Kaplan–Meier method, and the log-rank test was used to construct the curves

**Fig. 3:**
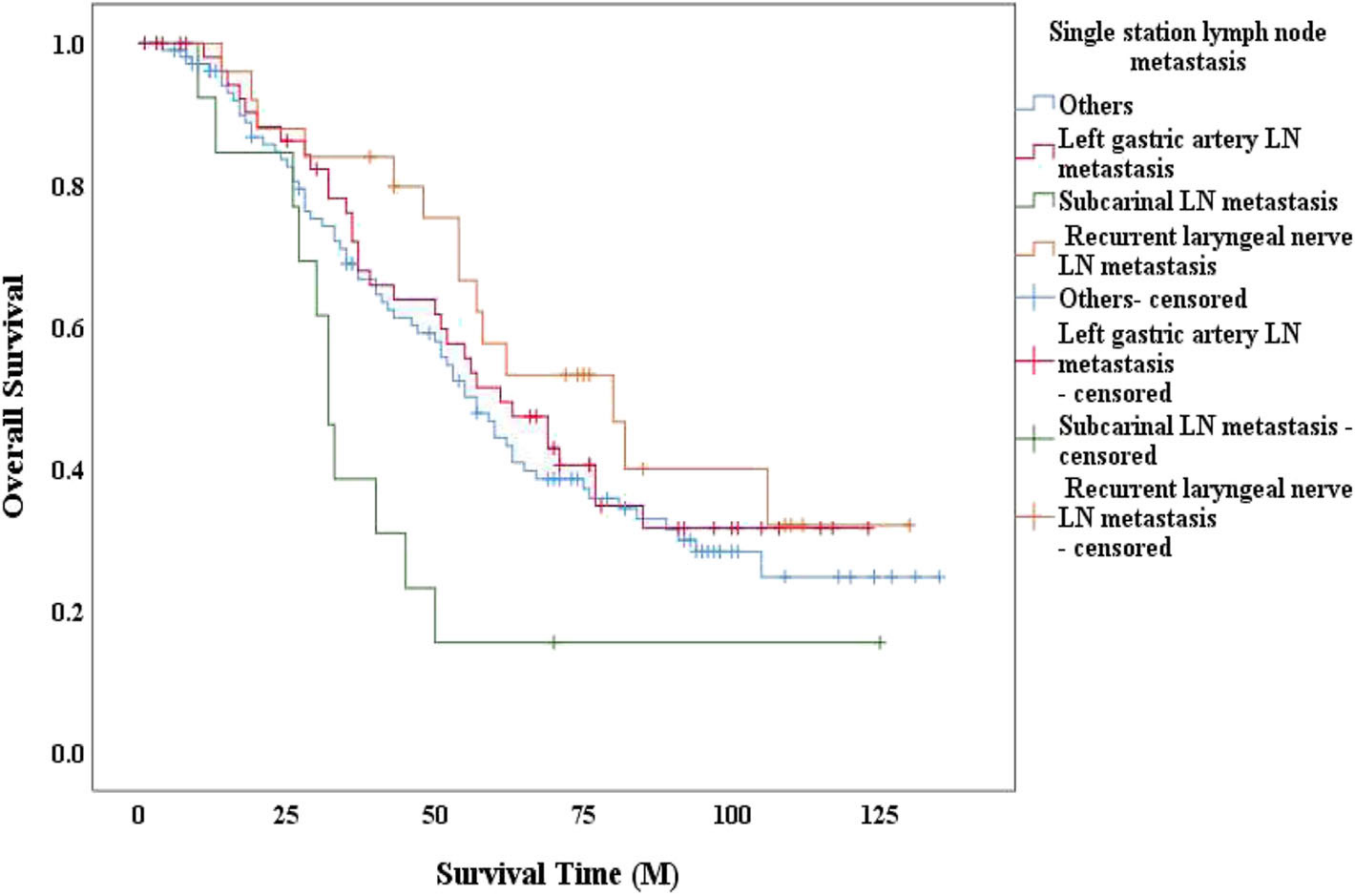
Overall survival among different single station LNM. Overall survival curves were used to explore the prognostic significance of different lymph nodes metastasis. Kaplan–Meier method, and the log-rank test was used to construct the curves. Single station LNM refers to only one lymph node metastasis. *P* value between left gastric artery LNM and subcarinal LNM is 0.014; *P* value between left gastric artery LNM and recurrent laryngeal nerve LNM is 0.417; *P* value between subcarinal LNM and recurrent laryngeal nerve LNM is 0.006

To demonstrate the prognostic significance of left gastric artery metastasis intuitively, we constructed CSS curves. CSS curves comparison between the groups with and without left gastric artery metastasis showed a significant difference with *P* <  0.001 (Fig. 2). We intend to show the prognostic difference among three different single stations LNM, we constructed the OS curves in patients with only one lymph node metastasis. The result showed patients with subcarinal LNM had the poorest survival compared to other two types. Moreover, results showed patients with left gastric artery LNM had a better survival than patients with the subcarinal LNM (*P* = 0.014, Fig. 3). Similar difference was found between the subcarinal LNM and the recurrent laryngeal nerve LNM (*P* = 0.006, Fig. 3).

**Fig. 4:**
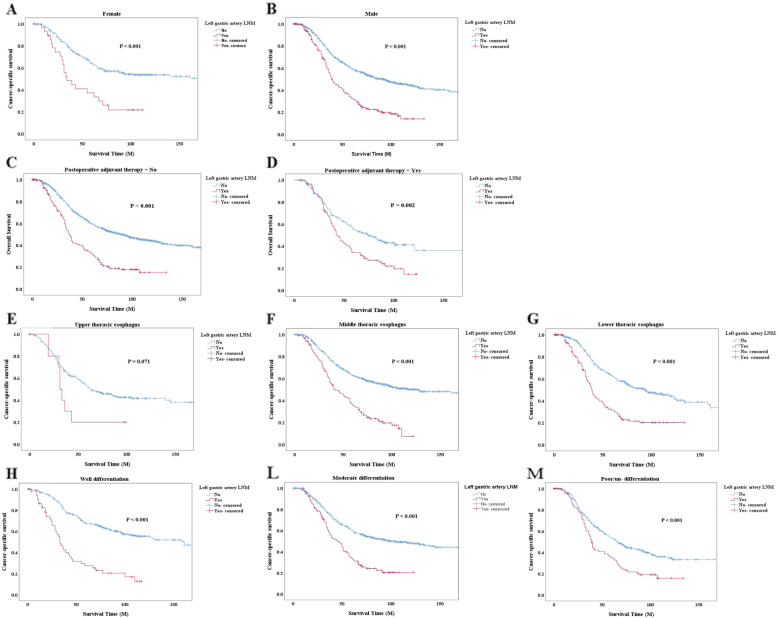
Cancer-specific survival in the subgroups on whether with left gastric artery LNM. Cancer-specific survival curves were used to compare the prognostic significance between the groups with and without left gastric artery metastasis. Kaplan–Meier method, and the log-rank test was used to construct the curves. **a** CSS in subgroups of male; **b** CSS in subgroups of female; **c** CSS in subgroups of without postoperative adjuvant therapy; **d** CSS in subgroups of with postoperative adjuvant therapy; **e** CSS in subgroups of middle thoracic esophagus; **f** CSS in subgroups of upper thoracic esophagus; **g** CSS in subgroups of lower thoracic esophagus; **h** CSS in subgroups of well differentiation; **l** CSS in subgroups of moderate differentiation; **m** CSS in subgroups of poor/un- differentiation

We conducted the stratified analyses to assess prognostic impact of left gastric artery LNM in three subgroups – sex, tumor location, differentiation and whether received postoperative adjuvant therapy. The results showed in the sexual and differential subgroups, survivals between whether with the left gastric artery LNM had a significant difference (*P* <  0.001, Fig. 4 a-b and h-m). Moreover, in the subgroups of tumor location, there were similar results when tumor located in middle or lower thoracic esophagus (*P* <  0.001, Fig. 4 f-g). Whereas there is no significant difference between the presence or absence of left gastric artery LNM, when tumor located in upper thoracic esophagus (*P* = 0.071, Fig. 4e). Furthermore, in the subgroups of whether received postoperative adjuvant therapy, the results were significant (*P* <  0.005, Fig. 4 c-d).

## Discussion

In this study, we addressed the prognostic role of LNM along the left gastric artery in postoperative patients with ESCC. Some previous studies showed that in patients without nodal involvement, the overall 5-year survival rate after surgical resection was between 70 and 92%, while this rate was only 18–47% among patients with LNM [18, 19]. Moreover, LNM was shown to be an accurate predictor of disease-free survival that can identify patients who may require adjuvant chemotherapy or chemoradiotherapy for the treatment of systemic metastases occurring after primary resection [19, 20]. Yang et al., [11] Mariette et al., [12] and Peyre et al. [13] reported that the number of positive LNs was an independent prognostic factor. An earlier study conducted by our team [17] suggested that thoracic/recurrent laryngeal nerve LN dissection could improve the overall and disease-free survival among ESCC patients. Feng et al. [16] revealed the importance of subcarinal LNM as an independent prognostic factor that predicted the site of metastatic LNs in ESCC. In clinical practice, LNM was often found to occur along the left gastric artery in patients with EC. Some investigators showed that LNM along the left gastric artery was correlated with prognosis in gastric cancer [21, 22]. However, LNM along the left gastric artery in EC has seldom been reported. At our clinic, we perform LN dissection during esophagectomy and regard LN dissection along the left gastric artery as a routine operation. Therefore, we aimed to explore the effect of the presence of LNM along the left gastric artery on the prognosis in patients after esophagectomy.

In this study, we found that sex, smoking history, drinking history, differentiation, vascular tumor thrombus, T stage, N stage, tumor location, and tumor length were significant variables for identifying patients with LNM along the left gastric artery (*P* <  0.05) (Table 3).

As mentioned before, studies on the prognosis in patients with LNM along the branches of the left gastric artery are rare. Furthermore, we determined the prognostic value of such metastasis among ESCC patients. We assessed factors associated with the prognosis in ESCC patients using univariate and multivariate Cox proportional hazards regression. After adjusting for age, tumor length, vascular tumor thrombus, N stage, T stage, and treatment, we found that LNM along the left gastric artery (*P* = 0.011, HR = 1. 920) was an independent prognostic factor in patients with ESCC. In the present study, we found that patients with LNM along the left gastric artery had poorer survival than those without metastasis, as shown by the CSS curve analysis (*P* <  0.001, Fig. 2). Our results clearly demonstrated that LNM along the left gastric artery could serve as an independent predictor of long-term survival among ESCC patients who have undergone surgery.

It is well known that LNM is correlated with tumor location in ESCC. To our knowledge, the farther the distance between the primary tumor location and the site of LNM, the poorer the prognosis in patients. The AJCC staging system is currently used in most countries and regions, in which N staging is based on the number of LNs with metastases. In many countries, particularly in Japan, some scholars currently recommend three field LN dissection during esophagectomy as a routine procedure. Three field LN dissection encompasses the cervical, thoracic, and abdominal LNs. These scholars found that the number of LNs resected correlated with N stage accuracy. Therefore, the 11th edition of the Japanese Classification of EC categorizes the N stage according to both the site and the number of metastatic LNs, regardless of the number of metastatic LNs (15). In our study, we also found that the number of resected total LNs was strongly associated with the LNM along the left gastric artery (*P* = 0.002, Table 3).

In the 11th edition of the Japanese Classification of EC, the station of the left gastric artery LN was termed as no.7, and the N stage differed according to the tumor location. Metastasis to the left gastric artery LN was defined as N3 in the upper thoracic esophagus, N2 in the mid-thoracic esophagus, and N1 in the lower thoracic esophagus. Abdominal LNM in thoracic EC was related to poor prognosis in clinical practice. Shimada et al. (23) revealed that patients with lower thoracic EC had a risk of perigastric LN metastases. We compared OS in patients with only one LNM, and patients with subcarinal LNM had the poorest survival compared to other two types. Moreover, results showed patients with left gastric artery LNM (*P* = 0.014, Fig. 3) or recurrent laryngeal nerve LNM (*P* = 0.006, Fig. 3), seems has a better survival than patients with the subcarinal LNM. Further research could be carried out to confirm.

Overall, our data showed a great significance in the relationship between the presence of LNM along the left gastric artery and poor prognosis. Our current results differed from those reported in the 11th Japanese Classification, which may be related to the small sample size of patients with LNs metastasis included in our study. Faced with the unsatisfactory results of the surgical outcomes at present, individualized treatment is encouraged under the concept of precision medicine, and further research using large sample data size needs to be conducted.

Our study has several limitations. It was a retrospective study, and there may be a lack of uniformity because although the data were all from a single institution, it included different pathologists and surgeons. Moreover, the range and number of LN dissections differed due to the operation time and the skill of the surgeon. Further studies are needed to explore the long-term effects of our results.

## Conclusions

In conclusion, LNM along the gastric artery was a predictive factor for long-term survival in patients who underwent esophagectomy for ESCC.

## Data Availability

Data available on request from the authors.

## Abbreviations

EC: Esophageal cancer
ESCC: Esophageal squamous cell carcinoma
LN: Lymph node
TLNs: Total lymph nodes
LNM: Lymph node metastasis
AJCC: American Joint Committee on Cancer
CSS: Cancer-specific survival
OS: Overall survival
HR: Hazard ratios
CIs: Confidence intervals
